# Giant keratocystic odontogenic tumor of the maxillary sinus and zygoma: A case report

**DOI:** 10.3892/ol.2014.2576

**Published:** 2014-09-29

**Authors:** JIANHUA ZHOU, LILI WANG, ZHENGGANG CHEN, JIANZHONG QIU, QUANJIANG DONG

**Affiliations:** 1 Department of Stomatology, Qingdao Municipal Hospital, Qingdao, Shandong 266071, P.R. China; 2 Central Laboratories, Qingdao Municipal Hospital, Qingdao, Shandong 266071, P.R. China

**Keywords:** keratocystic odontogenic tumor, zygoma, maxillary sinus

## Abstract

Keratocystic odontogenic tumors (KCOTs), formally known as odontogenic keratocysts, are benign developmental tumors that are found primarily in the mandibular molar region and ascending ramus. The disease is characterized by aggressive growth and a high recurrence rate following surgical treatment. The present study reports the rare case of a 25-year-old male with a giant KCOT involving the right zygoma, maxillary bone and maxillary sinus. The tumor was removed using a modified treatment of enucleation, grinding and cryotherapy. Recurrence has not been observed within the eight-month follow-up period. The present study discusses the clinical features and surgical management of this case.

## Introduction

Keratocystic odontogenic tumors (KCOTs) are benign tumors that comprise ~11% of all cysts of the jaws, worldwide (1–2). KCOTs are characterized by aggressive behavior and a high recurrence rate following surgical treatment. The disease commonly occurs between the ages of 10–30 years, predominantly in the mandibular molar region and ascending ramus (2). KCOTs are often asymptomatic, however, patients may occasionally present with swelling, pain and discharge (3–5). A number of therapies have been used in the management of KCOTs. Conservative treatments include simple enucleation, with or without curettage, decompression and marsupialization. Aggressive treatments, including peripheral osteotomy, cryotherapy with liquid nitrogen and resection, have also been reported (6). For the optimum management, it is necessary to assess the full extent of the lesion by a computed tomography (CT) scan. The present study reports the case of a patient with a giant KCOT involving the right zygoma and maxillary sinus, which was treated using a modified method (7,8). In this treatment modality, grinding of the peripheral bone and cryotherapy were used following enucleation in order to remove potentially remaining tumor tissue. Written informed consent was obtained from the patient.

## Case report

**Figure 1:**
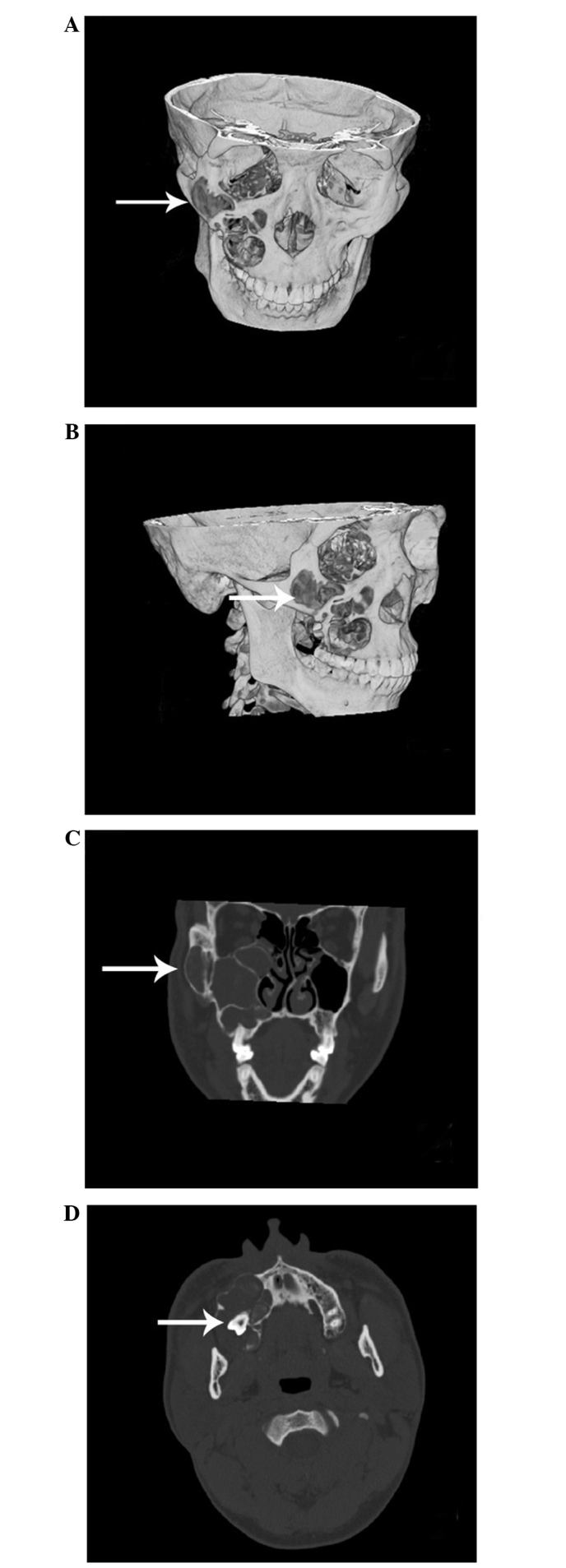
CT images. (A–C) Three-dimensional CT revealing a low density lesion located in the maxillary bone, zygomatic bone and maxillary sinus. (D) An axial scan revealing an impacted tooth. CT, computed tomography.

A 25-year-old male presented to the Qingdao Municipal Hospital (Shandong, China) with a two-month history of painless swelling on the right side of the face. The medical history of the patient was unremarkable. On clinical examination, a large swelling was identified on the right side of the face that was hard and bony on palpitation. A CT scan revealed a giant, multilocular and hypodense lesion of ~6.5×5.0 cm in size, with a well-defined margin (Fig. 1). The lesion involved the majority of the right maxillary bone, maxillary sinus and zygomatic bone. The third molar tooth was completely impacted (Fig. 1). Based on these observations, a tentative diagnosis of a cyst was proposed.

**Figure 2:**
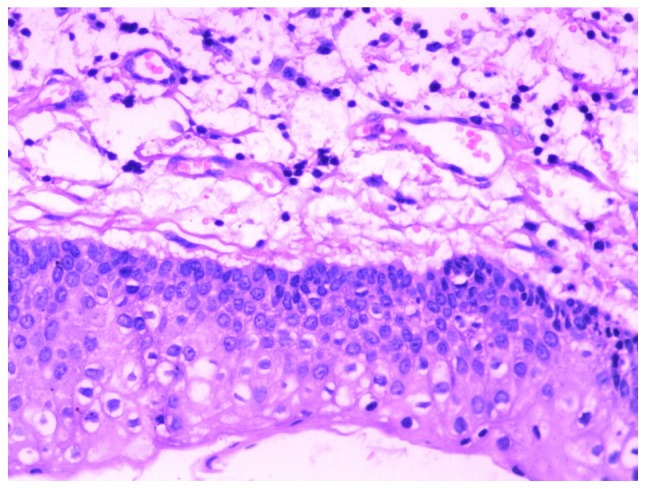
Photomicrograph revealing that the wall of the keratocystic odontogenic tumor was lined with a stratified epithelium and a corrugated keratinized lining (hematoxylin and eosin stain; magnification, ×200).

Under general anesthesia, the lesion was enucleated as a whole, using the Caldwell-Luc approach. The tooth that was associated with the lesion was simultaneously removed to avoid tumor fragmentation. Histological examination during the procedure identified a cystic tumor. The wall of the cyst was lined with a stratified squamous epithelium and a corrugated keratinized lining (Fig. 2). A diagnosis of KCOT was determined. To eliminate the possibility of residual cystic tissue, the margin surrounding the lesion was ground and then frozen with liquid nitrogen three times. Prior to the freezing of the surgical site, the adjacent tissues were protected using dry gauze pads. Subsequently, the patient exhibited a good recovery and no recurrence has been observed within the eight-month follow-up duration.

## Discussion

KCOTs are benign, unilocular or multilocular, but locally aggressive, developmental cystic neoplasms that were first described by Philipsen in 1956 (8). KCOTs are believed to arise from the dental lamina and are associated with impacted teeth. KCOTs can present as solitary or multiple lesions; the latter is usually one of the components of inherited naevoid basal cell carcinoma syndrome. PTCH, a tumor suppressor gene located on chromosome 9q22.3-q31, is associated with the development of KCOTs (5). This gene is part of the Hedgehog signaling pathway and has been revealed to be associated with several epithelial tumors, suggesting that the disease is neoplastic in nature (4).

Clinically, the majority of patients with KCOTs present with pain, swelling, a mass with discharge or aggressive growth or localized asymptomatic swelling. KCOTs occur most frequently in the mandible, accounting for 65–83% of cases. Overall, <1% of KCOTs occur in the sinus (9). In the present case, the involvement of the giant KCOT with the majority of the zygoma and maxillary sinus was unusual. To the best of our knowledge, this is the only reported case in the English literature. KCOTs most often grow along the anterior-posterior direction within the jaws, without causing evident bone expansion, leading to the delayed appearance of symptoms and clinical signs (10). Thus, the tumor may reach a size as large as that reported in the current study. In addition, the extremely large size and the involvement of the zygoma suggest that there was aggressive expansion of the tumor in the present case.

Although various treatment modalities have been used in the management of KCOTs, the most effective treatment remains controversial. Blanas *et al* (11) reviewed treatment options for KCOTs, including simple curettage, enucleation, marsupialization and resection. All of these treatments may lead to recurrence, with the exception of resection (12). In previous years, there has been a tendency to use decompression and irrigation to prevent recurrence of the disease. The benefits of this technique are due to the minimal surgical morbidity and the decreased damage to the associated structures (13). In the present case, the cystic tumor was extremely large in size and invaded the majority of the zygomatic-maxillary complex. Therefore, the modality of decompression of the cyst was not selected due to the lower repair capacity of the maxillary sinus compared with the other maxillofacial bones. A modified treatment was used in the present case to prevent the recurrence of the tumor. Following conventional enucleation, the thin bone around the lesion was ground with a drill and frozen with liquid nitrogen to eliminate potential residual cystic tissue or satellite microcysts. In contrast to Carnoy’s solution, liquid nitrogen can induce cell necrosis and preserve inorganic bone structures, which destroys osteogenic and osteoconductive properties (14). This cryotherapy technique preserves the bone framework and leads to improved repair and bone height recovery. In the eight-month follow-up period, no recurrence of the KCOT was observed. A long-term follow-up, however, is required.

In conclusion, KCOT involving the zygoma, usually caused by a large tumor size, is extremely rare. In the present case, a modified treatment with enucleation, grinding and cryotherapy was demonstrated to be an effective treatment for KCOT. Resection of a large-sized tumor may not be adequate as it may cause extensive tissue destruction. The results of the present study indicate that modified treatment may present a reasonable treatment option for giant KCOTs.
